# The role of fibroblast growth factor 1 and 2 on the pathological behavior of valve interstitial cells in a three-dimensional mechanically-conditioned model

**DOI:** 10.1186/s13036-019-0168-1

**Published:** 2019-05-27

**Authors:** Ngoc Thien Lam, Ishita Tandon, Kartik Balachandran

**Affiliations:** 1 Cell and Molecular Biology Program, University of Arkansas, Fayetteville, AR 72701 USA; 2 Department of Biomedical Engineering, University of Arkansas, 122 John A. White Jr. Engineering Hall, Fayetteville, AR 72701 USA

**Keywords:** Fibroblast growth factor, Fibroblast growth factor receptor, Valve interstitial cells, Three-dimensional cell culture, Mechanical stretch, Heart valve disease

## Abstract

**Background:**

More than five million Americans suffer from heart valve disease annually, a condition that worsens cardiac function and gradually leads to heart failure if appropriate treatment is not performed on time. Currently no medication can cure heart valve disease, leaving surgical intervention as the only viable option for patients at late stages of cardiac valve disease. Tremendous efforts have been undertaken to elucidate how resident cells in the valves respond to pathological stimulation as well as the underlying mechanisms that regulate these responses, to identify potential therapeutic targets for non-surgical treatment of valvular heart disease.

**Results:**

Cardiac valve interstitial cells (VICs) naturally reside in a complex three-dimensional environment under varying hemodynamics, which is difficult to replicate in vitro*.* As a result, most cell signaling studies in the field have traditionally been conducted on two-dimensional models or in the absence of hemodynamic forces. Previously, we reported the fabrication of a hydrogel scaffold that could be used to culture valve cells under dynamic mechanical stimulation in a valve-mimetic environment. This model, therefore appeared to be suitable for VIC signaling studies as it provided cells a three-dimensional environment with the ability to incorporate mechanical stretching stimulation. Utilizing this model, we investigated the possible role of fibroblast growth factor 1 and 2 (FGF1 and FGF2) via FGFR1 receptor signaling in regulating valve cell activation under physiological (10% stretch) and pathological (20% stretch) mechanical conditions as well as in mediating cell proliferation and metabolism via the Akt/mTOR pathways. We reported that 1) FGF1 and FGF2 treatment was able to maintain the quiescent phenotype of VICs; 2) Cells increased proliferation as determined by optical redox ratios under elevated cyclic stretch via Akt/mTOR pathways; and 3) FGF1 and 2 signaling via the FGFR1 reduced VIC proliferation and activation under elevated cyclic stretch conditions.

**Conclusions:**

Overall, these results suggested that targeting FGFR1 receptor signaling may represent a possible therapeutic strategy for preventing heart valve disease progression.

**Electronic supplementary material:**

The online version of this article (10.1186/s13036-019-0168-1) contains supplementary material, which is available to authorized users.

## Background

As the heart contracts and relaxes, its valves elegantly open and shut, maintaining unidirectional flow of blood. Throughout this dynamic process, resident valve cells actively remodel and maintain homeostasis via an intricate system of signaling networks between cells and their microenvironment. Malfunction of valve function due to disrupted homeostasis is associated with impaired cardiac function and heart valve disease [1]. To prevent long-term damage to the heart, surgical intervention to replace heart valves is a must as currently there are no drug therapies to halt or reverse disease progression [2]. Approximately, 67,500 aortic valve replacement procedures are performed every year in the U.S [3]. Consequently, researchers have sought to understand the cellular and molecular processes that underlie valve disease pathogenesis, hoping that it might possibly lead to nonsurgical treatment.

The study of pro-fibrotic mechanisms has been one area of focus in valve pathology. In static, two-dimensional (2D) culture conditions, Xu et al reported the association of serotonin-mediated pro-fibrotic signaling with valve disease. Specifically, treatment of valve interstitial cells (VIC) with serotonin reportedly caused up-regulation of transforming growth factor-β1 (TGF-β1) activity which in turn caused increased synthesis of extracellular matrix (ECM) proteins (collagen and GAGs) as seen in heart valves of carcinoid syndrome patients [4, 5]. It was also reported that the serotonin-2A receptor subtype (5HTR2A) was involved in 5HT up-regulation of active TGF-β [5]. Similarly, at the tissue level, Balachandran et al subjected aortic valve cusps to elevated cyclic stretch and reported the up-regulation of 5HTR2A and 5HTR2B expression which was associated with increased proliferation and ECM production in response serotonin addition [6]. TGF-β signaling activated VICs from a quiescent fibroblastic phenotype to a contractile myofibroblast-like phenotype, and was a key regulator of wound repair by VICs [7]. Using a scratch wound model, a study showed that VICs at the wound edge produced TGF-β, which then enhanced wound repair through increasing cell activation, proliferation, wound repair and formation of stress fibers. Earlier studies reported the presence of TGF-β within calcific stenosis cusps that mediated the calcification of aortic VICs in culture through mechanisms involving apoptosis [8]. Interestingly, Cushing et al reported that fibroblast growth factor 2 (FGF2) effectively blocked TGF-β1-mediated myofibroblast activation and also the development of pathological contractile and calcifying phenotypes in aortic valvular interstitial cells [9]. Similarly, FGF2 was found to promote VIC wound repair through inhibition of the TGF-β/Smad-2/3 signaling pathway [10]. Treatment with FGF2 was able to reduce myofibroblast activation in porcine VICs [9]. Further, the possible protective role of FGF2 on VICs has been tested directly in cell culture media and showed that FGF2-containing cell culture media was able to maintain and dedifferentiate the VICs to a quiescent, fibroblastic phenotype with phenotypic and functional characteristics ascribed to cells in the intact valve [11].

While these results suggest that the maintenance of FGF2-mediated signaling pathways is integral for the prevention of deleterious fibrosis in heart, the role of FGF1 and FGF2 in valve pro-fibrotic signaling is not well-understood. Additionally, as both FGF1 and FGF2 belong to the FGF1 subfamily and FGF1 has been demonstrated to be able to improve cardiac functional recovery and enhance cell survival after ischemia and reperfusion [12, 13], it would be significantly meaningful for the heart valve field to investigate and elucidate the pathophysiological role of FGF1 in valve cells in comparison with FGF2. It is also worth noting that most benchtop studies on the pathophysiology of heart valve interstitial cells have been done at the 2D scale or using ex vivo leaflet explants, but not at 3D cellular scale with appropriate in vivo- simulated mechanical conditions. Therefore, in order to fill in this research gap, we proposed to use a mechanically robust 3D hydrogel platform [14] to study FGF signaling in the presence of physiological or pathological mechanical conditions. There have been numerous studies in other fields that suggested the involvement of Akt and mTOR signaling as a mediator of FGF signaling in many biological processes [15–18]. We thus hypothesized that FGF pathway activation in VICs is mediated by Akt/mTOR signaling. Our study provides fundamental insights into valve cell pathophysiology under abnormal mechanical stretch and suggests the potential of FGF1/FGF2 as targets for drug therapies for the treatment of heart valve disease.

## Methods

### Immunohistochemistry of non-diseased and diseased human heart valve leaflets

We first examined the expression of fibroblast growth factor 1 and 2 (FGF1 and FGF2) and their receptors in non-diseased and diseased heart valve leaflets. Four de-identified IRB-exempt paraffin-embedded blocks of human aortic valve leaflets were obtained from the University of Arkansas for Medical Sciences tissue bank. Demographic and sex information of the samples were not known. Samples were divided into two categories depending on the presence of calcific lesions on the tissues, namely (I) non-calcified (*n* = 3), (II) calcified (*n* = 1). Sections were then immunostained using standard protocols against the following proteins: FGF1, FGF2, FGFR1, and FGFR2. Briefly, after deparaffinization and rehydration, sections were incubated in 10 mM citrate buffer, pH 6 at 95 °C for 10 min for antigen retrieval. The slides were then allowed to cool at room temperature for 20 min, followed by rinsing twice in Dulbecco’s phosphate buffered saline (dPBS, Gibco), 5 min each. Blocking was performed using either 20% goat serum or donkey serum (Life Technologies) in dPBS for 1 h at 37 °C. Slides were incubated with either anti-FGF1 (Santa Cruz Biotechnology, 1:10), anti-FGF2 (Santa Cruz Biotechnology, 1:10), anti-FGFR1 (Abcam, 1:25), or anti-FGFR2 (Abcam, 1:25) antibodies, with 2% goat or donkey serum in a humid chamber at room temperature for 2 h. Following primary antibody incubation, the slides were washed in PBS three times, 5 min each. The slides were then incubated with 1:100 secondary antibody (Alexa Fluor 488 or Alexa Fluor 594), 2% goat or donkey serum and 4′,6-diamidino-2-phenylindole (DAPI) for 1 h at room temperature in a humid chamber, protected from light, followed by washing in dPBS three times. A drop of Prolong Gold (Life Technologies) anti-fade mounting media was used to coverslip the sections. Clear nail polish was used to seal the edges of the coverslip. Slides were then imaged using a standard epifluorescence microscope (Nikon Ti).

### Valve interstitial cell isolation and culture

Valve interstitial cells for in vitro culture were isolated from fresh porcine hearts obtained from a local abattoir (Cockrum’s Custom Meat Processing and Taxidermy, AR) using techniques published by us previously [14, 19, 20]. Briefly, the heart was transported to the laboratory in ice-cold, sterile PBS solution and quickly dissected in the laboratory using aseptic techniques. All three aortic valve leaflets were dissected and incubated in 1 mg/ml collagenase solution (Worthington, NJ) for 3 h at 37 °C with frequent agitation. After collagenase digestion, cold 10% fetal bovine serum (FBS)-containing Dulbecco’s Modified Eagle Media (DMEM) was added to arrest enzymatic activity. The solution was filtered with the cell strainer 100 μm pore size (Corning, NY) to remove any remaining tissue debris prior to centrifugation for 5 min at 200 g and 4 °C. The resulting cell pellet was re-suspended in 10% FBS-containing cell culture media, plated in a flask and maintained in a 37 °C incubator. Fresh media was changed at least every 3 days. Cells from passage 1–7 were used in all subsequent two-dimensional (2D) and three-dimensional (3D) culture studies.

### Indirect immunofluorescent staining of valve interstitial cells

We tested the direct effects of FGF1/FGF2 on valve cell phenotype in 2D culture. Valve interstitial cells were seeded onto glass coverslips at 500,000 cells per coverslip. Upon reaching confluency, they were divided into three groups which were supplemented with three different culture media formulations, named as 10% FBS-containing media, FGF1 media and FGF2 media (both Peprotech, NJ), respectively. Specific reagents for these media formulations are listed in Table 1. Cells were maintained in culture for 2 weeks, with fresh media changes every two to 3 days. At the end of the second week (day 14), coverslips were fixed and stained with common phenotypic markers of VICs. Briefly, 4% paraformaldehyde (PFA) and 0.5% Triton X-100 were used to fix and permeabilize the cells prior to 1 h blocking in 5% bovine serum albumin (BSA). Primary antibodies were added and incubated overnight, including α-SMA (differentiation/activation marker for smooth muscle cells - 1:200), vimentin (intermediate filament protein - 1:1500), calponin (smooth muscle cell marker - 1:1000), Ki67 (nuclear protein associated with cell proliferation - 1:500) and osteopontin (bone structural protein and indicator of osteogenic differentiation in VICs - 1:200) (all from Abcam). The next day, appropriate secondary antibodies and DAPI (1:200) (to stain the nuclei) were added. These coverslips were then mounted onto glass slides and imaged. To obtain a quantitative measure of cell proliferation, Ki67-positive cells were manually counted and normalized with the total number of DAPI-positive cells in each image field. This sample preparation was repeated for a separate set of samples for two-photon excited fluorescence imaging. The image acquisition process is described in the redox imaging section.

**Table 1:** List of supplements used for the cell culture medium formulations

	FGF1 medium	FGF2 medium	10% FBS-containing medium
FGF1 or FGF2	50 ng/ml FGF1	50 ng/ml FGF2	N/A
FBS	2%	2%	10%
Antibiotics/antimycotics	1%	1%	1%
HEPES	1%	1%	1%
Insulin	50 ng/ml	50 ng/ml	N/A

### Pharmacological inhibition of fibroblast growth factor receptor subtype 1 (FGFR1)

The selective inhibitor of the FGF1 receptor tyrosine kinase, PD166866 (Sigma), was used to block FGFR1 [21], to study the involvement of FGF1/FGF2 in mediating downstream cellular responses. For this purpose, we first tested FGFR1 inhibition on a 2D monolayer of VICs. Western blotting and standard 3-(4,5-dimethylthiazol-2-yl)-2,5-diphenyltetrazolium bromide (MTT) proliferation assays were carried out to first determine the appropriate working concentrations of this inhibitor on VICs. Cells were serum-starved overnight before the experiment. A total of six different concentrations of PD166866 were tested: 1, 10, 50, 100, 500 and 1000 nM. Cells were incubated with the inhibitor for 2 h, following which, 50 ng/ml of FGF1 or FGF2 was separately administered to the cells for 10 min before protein extraction took place using RIPA lysis buffer. Control samples included cells that received only FGF1 or FGF2 treatment without PD166866, cells that received only PD166866 at the highest dose, or cells that did not receive any treatment. A bicinchoninic (BCA) assay was carried out to determine protein concentration, followed by western blotting to examine the effect of FGFR1 inhibitor doses on Akt phosphorylation. The concentration that gave the least signal of phosphorylated Akt was chosen for the subsequent experiments.

The western blotting protocol was briefly as follows. Criterion 10% polyacrylamide gels (Bio-Rad) were loaded with at least 10 μg of protein and subject to electrophoresis for 1 h at 150 V constant voltage. The gel was transferred to PVDF membranes for western blotting analysis. Membranes were blocked for 2 h at room temperature with blocking buffer (LiCor), and probed with phosphorylated-Akt antibody (Cell signaling, 1:200) and β-actin (Abcam, 1:200) and incubated overnight at 4 °C. The next day, these membranes were washed and incubated with appropriate secondary antibody (Licor) and imaged using a Licor Odyssey scanner. These membranes were stripped with stripping buffer (Licor) and re-probed with total Akt antibody (Cell signaling, 1:100) prior to incubation with appropriate secondary antibody and imaged again. Signaling was quantified by comparing the band intensity obtained for the phosphorylated protein and normalizing it with the intensity obtained for total protein. β-actin was used as an additional loading control.

For the MTT assay, VICs were plated in a 96-well plate. All treatment conditions were the same as above. After addition of inhibitor and ligands, cells were maintained in culture for a further 3 days to account for the longer timescale of proliferation [22], compared to the transient expression of receptor-mediated phosphorylation. At the end of the third day, cell media was aspirated and 5 mg/ml of MTT were added to the cells and incubated for 4 h at 37 °C until insoluble purple formazan crystals formed as the result of enzymatic activity in active dividing cells. MTT solution was removed and DMSO was added to dissolve the crystals. Absorbance measurements were then taken at 570 nm. This absorbance reading was proportional to the proliferation capacity of the cells, and was normalized against a blank sample which had only DMEM-phenol red free, MTT and DMSO.

### Cyclic mechanical stretching of valve interstitial cells in a three-dimensional (3D) hydrogel

VICs exist in a 3D extracellular matrix environment, and undergo continuous mechanical stimulation in vivo. We therefore utilized a 3D matrigel-collagen substrate, with cyclic mechanical stretching to better simulate the in vivo environment of the valve, for all subsequent experiments. The matrigel-collagen hydrogel, its fabrication and subsequent cyclic stretching, has been reported by us previously [14]. Briefly, 1 million VICs were mixed with a 50–50 ice-cold mixture of matrigel-collagen hydrogel (1 mg/mL final collagen concentration), and allowed to polymerize at 37 °C for 30 min. Cell culture medium was added and the hydrogel constructs kept in culture overnight to allow for the cells to attach to the matrix. The next day, these constructs were stretched uniaxially at either 0, 10% or 20% peak cyclic stretch, which represented static, healthy or pathologic mechanical conditions, respectively. Validation of these stretch magnitudes, and the negligible variation in strain across the thickness of our hydrogel samples, was reported in our previous publication [14].

### Quantification of Akt and mTOR phosphorylation following cyclic mechanical stretching

**Fig. 1:**
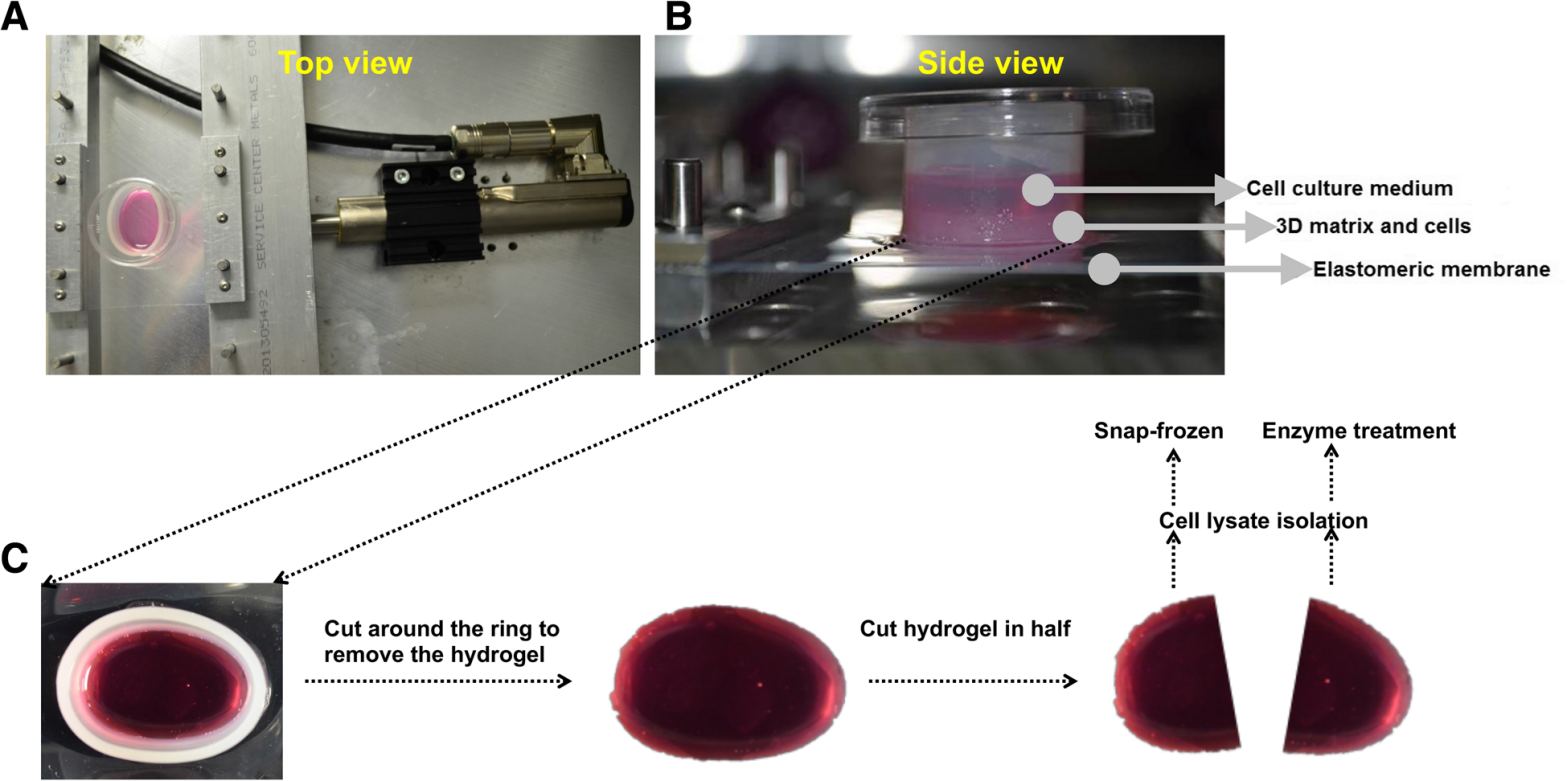
Schematic diagram showing the **a** top and **b** side views of the cyclic stretch culture bioreactor used in this study. **c** Schematic of the methods to collect cell lysate for phosphorylated and total protein analysis in this study

We first quantified Akt and mTOR phosphorylation following the above three different magnitudes of cyclic mechanical stretch. Five different time points were examined, namely, 10 min, 1 h, 6 h, 24 h and 48 h after initiation of stretch, to account for the transient expression of phosphorylated Akt and mTOR expression. For all subsequent western blotting analysis, the hydrogel sample was first cut in half and processed in two different ways for protein collection (Fig. 1). Immediately, after the stretching was stopped at a certain time point, samples were taken out of the stretcher device, washed briefly with PBS and divided into half with a scalpel. One half of the sample was immediately snap-frozen after stretch termination and RIPA lysis buffer was added. The solution was vortexed, centrifuged and supernatant was collected. This lysate was used to detect the transient expression of phosphorylated proteins. The second half of the hydrogel construct was treated with 1 mg/ml of collagenase-1 and dispase-2 for 1 h at 37 °C to degrade the hydrogel materials, thus releasing cells from the matrix. RIPA lysis buffer was then added to the cells for protein collection. This lysate was used to detect the stable expression of total protein. BCA assay were then carried out to determine protein concentration.

For western blots, criterion 4–15% polyacrylamide gels (Bio-Rad) were loaded with at least 20 μg of protein and subjected to electrophoresis for 1 h at 150 V constant voltage. The gel was transferred to PVDF membranes for western blotting analysis. Membranes were blocked for 2 h at room temperature with blocking buffer (LiCor), and probed with appropriate primary antibodies and incubated overnight at 4 °C. In particular, membranes that had lysates from snap-frozen samples were probed with antibodies for phosphorylated-proteins, namely, phosphorylated-Akt (Cell signaling, 1:200), or phosphorylated-mTOR (Abcam, 1:500), while membranes with enzymatically treated lysates were probed with antibodies for total proteins, namely, total Akt, or total mTOR (Cell signaling, 1:100). After overnight incubation, these membranes were washed and incubated with appropriate secondary antibody (Licor) and imaged using a Licor Odyssey scanner. Protein expression was quantified by comparing the band intensity obtained for the phosphorylated protein and normalizing it with the intensity obtained for total protein. β-actin was used as an additional loading control. The time point that yielded the strongest phosphorylation signal was then chosen for subsequent experiments.

### Quantification of Akt and mTOR phosphorylation in stretched valve interstitial cells with FGF1 or FGF2 stimulation

For this experiment, a total of 13 different experimental conditions were examined (Table 2). 3D matrigel-collagen hydrogel constructs were prepared as described earlier, and in our previous publication [14]. After overnight culture, for samples that were treated with inhibitor, FGFR1 inhibitor was added to the samples for 24 h, at a concentration determined by the previous western blot and MTT assays. FGF1 or FGF2 was then added to the cells for 1 h, followed by stretching at 10% or 20% (time point determined by experiments in previous section). For samples that did not receive inhibitor treatment, FGF1 or FGF2 was added directly to the samples for 1 h before the stretch initiation. Protein collection and western blot were performed as described above.

**Table 2:** List of all treatment conditions tested

Sample types	Without PD166866	With PD166866
10% stretch	Stretch only	+ PD166866
	+ FGF1	+ PD166866 + FGF1
	+ FGF2	+ PD166866 + FGF2
20% stretch	Stretch only	+ PD166866
	+ FGF1	+ PD166866 + FGF1
	+ FGF2	+ PD166866 + FGF2
No stretch	No inhibitor or FGF treatment	

### Two-photon excited fluorescence to determine optical redox ratio in valve interstitial cells

Separate samples were prepared with the same FGF and/or inhibitor conditions as described in Table 2. For these experiments, as we were analyzing acute cell metabolism, the samples were allowed to stretch continuously for 24 h before data acquisition to ensure cells have sufficient time to proliferate and become metabolically active [20]. Before imaging, the cell culture chamber was placed carefully in a heated chamber to maintain cells at 37 °C throughout the imaging procedure. Images were acquired using a custom-built resonant-scanning multiphoton microscopy platform with a 20X, 0.8 NA water immersion objective (Nikon, Japan) and a MaiTai ultrafast Ti:Sapphire tunable laser source (Spectra-Physics, Santa Clara CA) [20]. The laser excitation source was tuned to 750 nm (NADH fluorescence), 860 nm (FAD fluorescence) and 800 nm (Collagen). Laser power was kept constant throughout the experiment and the photomultiplier tube (PMT) gain was fixed at 90%. Images of a cuvette filled with 4 ng/mL Rhodamine B (Sigma) were acquired via a 607 nm/70 nm bandpass filter, under identical conditions for both NADH and FAD acquisitions, to normalized for possible day-to-day variation in laser intensity. Images were analyzed using a custom MATLAB script and optical redox ratio was calculated using the following equation on a per pixel basis [23]:

$$ \mathrm{Redox}\kern0.5em \mathrm{ratio}=\frac{\left[ FAD\right]}{\left[ NADH\right]+\left[ FAD\right]} $$

[FAD] represents the intensity of the FAD image normalized by the corresponding rhodamine intensity. [NADH] represents the intensity of the NADH image normalized by the corresponding rhodamine intensity. After imaging, the samples were lysed with RIPA buffer and analyzed via BCA assay and western blot to detect the expression of phenotypic markers of VICs (i.e. calponin and α-SMA) and expression of heat shock protein 47 (hsp47).

### Statistical analysis

All quantitative data were first analyzed for normality using the Anderson-Darling method. All normally distributed data were subsequently analyzed by two- or three-way ANOVA followed by Holm-Sidak multiple pairwise comparisons. A *p*-value of less than 0.05 was used to indicate statistical significance differences between samples. Data was plotted as mean with standard error bars. Sample sizes for each experiment result are indicated in the respective figure captions.

## Results

### FGF1 and FGF2 promoted cell proliferation in two-dimensional valve interstitial cell culture

**Fig. 2:**
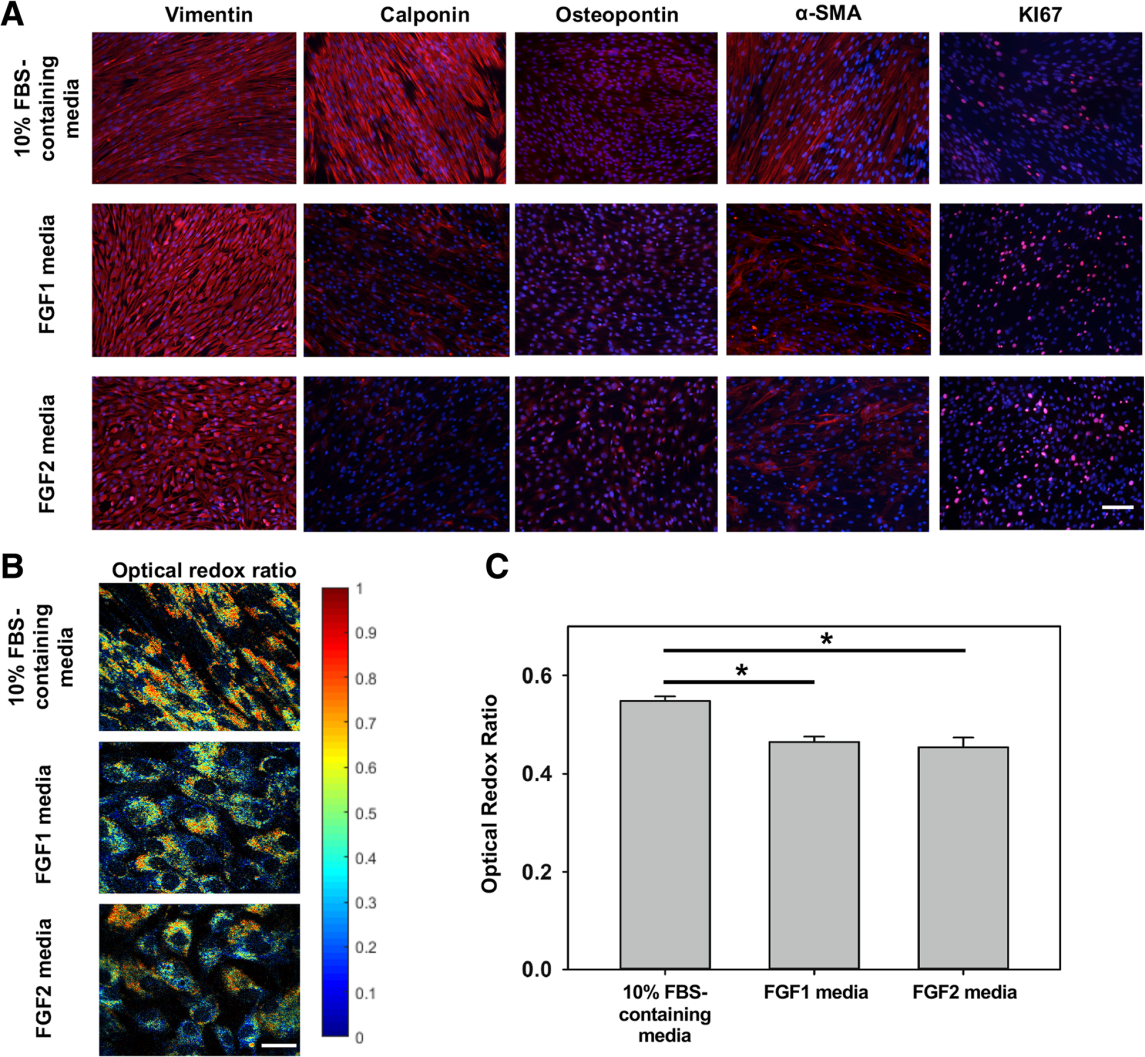
Immunofluorescence staining images for different proteins expressed when VICs were cultured in 3 different media, scale bar =100 μm (**a**), and optical redox ratio, scale bar = 10 μm (**b**). Quantification of optical redox ratio images (**c**). *n* = 5, * *p* < 0.05

FGF2 has been previously used in 2D VIC culture to maintain the cells in a quiescent, fibroblast-like state [11]. We were thus first interested in comparing the effects of FGF1 and FGF2 on VIC phenotype, using 10% FBS-containing media as control. Immunofluorescence showed that cells in FGF1 and FGF2 media behaved in a different way compared to cells in 10% FBS-containing media (Fig. 2). Particularly, VICs expressed vimentin, a marker known to be highly expressed in quiescent VICs [11, 24], at a higher expression level in FGF1 and FGF2 medium than in 10% FBS-containing medium, while calponin was strongly expressed when cells were cultured in 10% FBS-containing medium as compared to FGF1 and FGF2 media. Other activated VIC markers, α-SMA and osteopontin, showed comparable expression among 3 media. VICs also showed increased propensity for proliferation in the presence of FGF1 and FGF2 media as evidenced by Ki67 stained images. Normalized proliferation index (Additional file 1: Figure S1A), was significantly higher (*p* < 0.05) in the FGF1 and FGF2 media compared to 10% FBS-containing media. This observation was further supported by measurement of the optical redox ratio of NADH/FAD which correlates to the cellular metabolic activity [20]. Redox ratio was significantly decreased (*p* < 0.05) in cells that were cultured in FGF1 and FGF2 media compared to cells that were maintained in 10% FBS-containing media. When comparing between FGF1 media versus FGF2 media, no significant differences were observed. Additionally, we observed a negative correlation (*r* = − 0.981, *p* = 0.12) between normalized proliferation index and optical redox ratio (Additional file 1: Figure S1B). Overall, these observations suggested the possible role of FGF1 and FGF2 in modulating VIC phenotype such that cell proliferation was promoted, while expression of activated phenotypic markers were reduced.

### FGF1 and FGFR1 were moderately expressed in non-calcified and calcified aortic valves while FGF2 was strongly expressed in calcified aortic valves

**Fig. 3:**
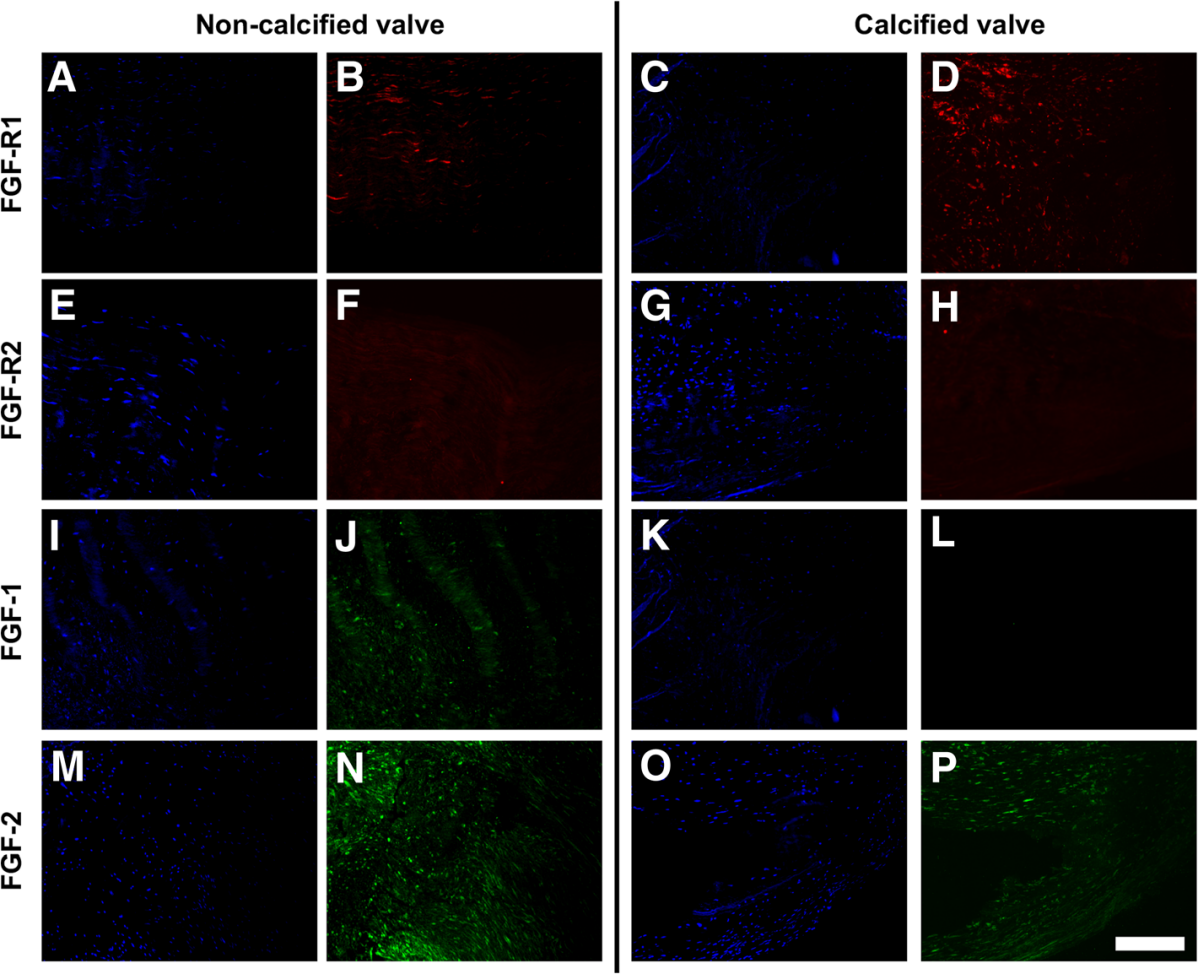
Immunohistochemistry staining of FGF-R1 (**a**, **c**: DAPI; **b**, **d**: marker), FGF-R2 (**e**, **g**: DAPI; **f**, **h**: marker), FGF-1 (**i**, **k**: DAPI; **j**, **l**: marker), and FGF-2 (**m**, **o**: DAPI; **n**, **p**: marker) for non-calcified and calcified valve leaflets. Scale bar = 50 μm

Immunohistochemistry of human aortic valve leaflets showed expression of FGFR1 (Fig. 3a – d) in both non-calcified and calcified valves while FGFR2 expression was low for all samples (Fig. 3e – h). This result consequently led us to a decision to target FGFR1 in the following inhibitor studies. Furthermore, FGF1 expression (Fig. 3i – l) was high in healthy leaflets and low in calcified leaflets, while FGF2 expression (Fig. 3m – p) was high in healthy samples and calcified leaflets. These observations suggested a possible correlation between FGF receptor signaling and presence of calcific disease.

### Elevated stretch upregulated Akt and mTOR phosphorylation in valve interstitial cells

**Fig. 4:**
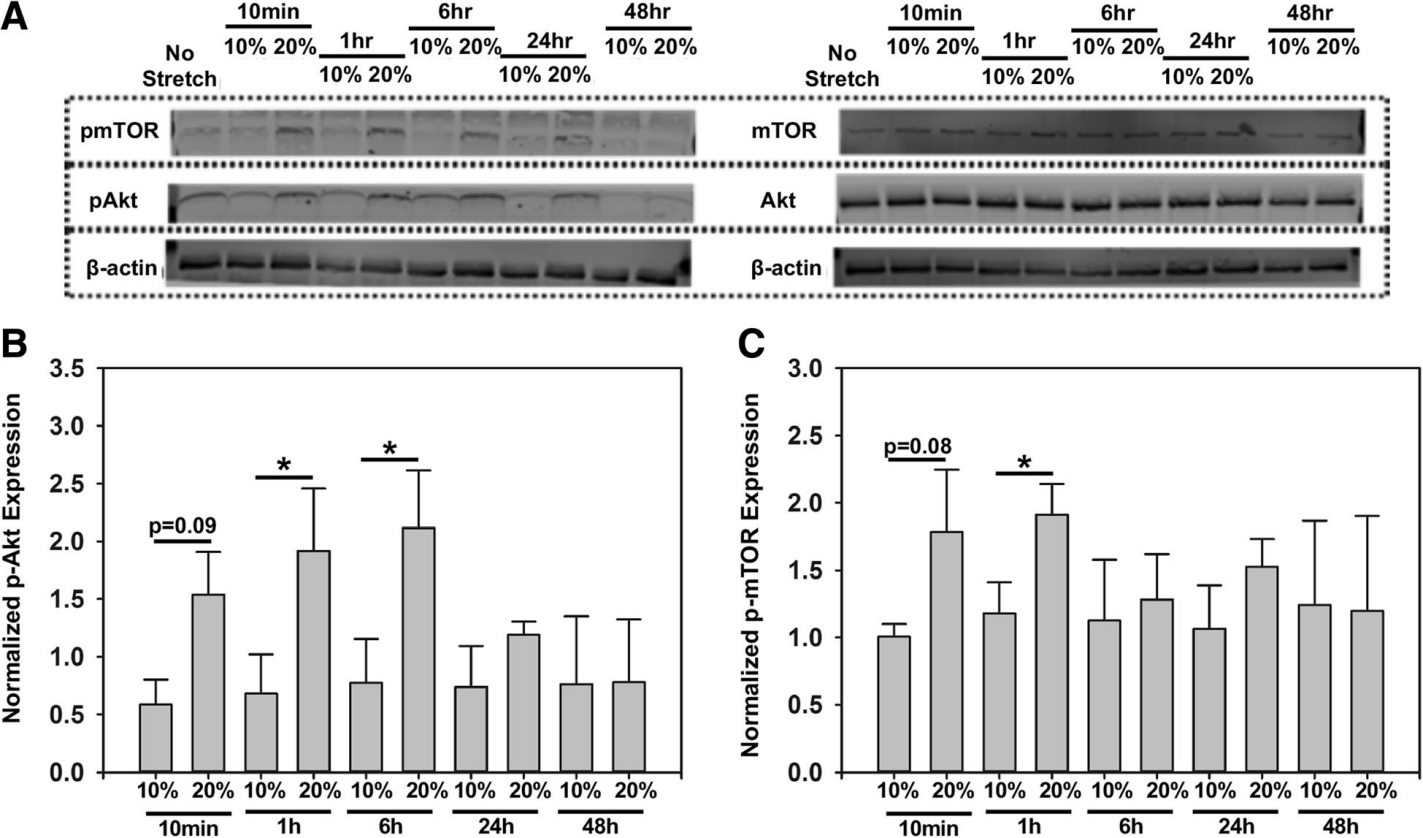
Representative western blot (**a**) and semi-quantitative analysis of Akt (**b**), and mTOR (**c**) phosphorylation at different time points, *n* = 3, *p < 0.05

We next investigated the effect of normal and elevated cyclic stretch on transient Akt and mTOR phosphorylation in VICs. VICs were cultured in a 3D matrigel-collagen hydrogel and subjected to 10% or 20% uniaxial cyclic stretch to simulate physiological and pathological conditions, respectively. Unstretched samples served as controls. As shown in western blot analyses (Fig. 4a), the expression of phosphorylated Akt (Fig. 4b) and phosphorylated mTOR (Fig. 4c) was higher at elevated stretch (20%) compared to normal stretch (10%). The data suggested that the Akt signaling was significantly increased (*p* < 0.05) at an elevated cyclic stretch magnitude (20%) at the 1 h and 6 h time points, while mTOR signaling was significantly increased (*p* < 0.05) at the 1 h time point at elevated stretch (20%). Based on these results, we chose the 1 h time point for subsequent Akt and mTOR phosphorylation experiments.

### Inhibition of FGFR1 receptor signaling significantly reduced Akt and mTOR phosphorylation in valve interstitial cells

**Fig. 5:**
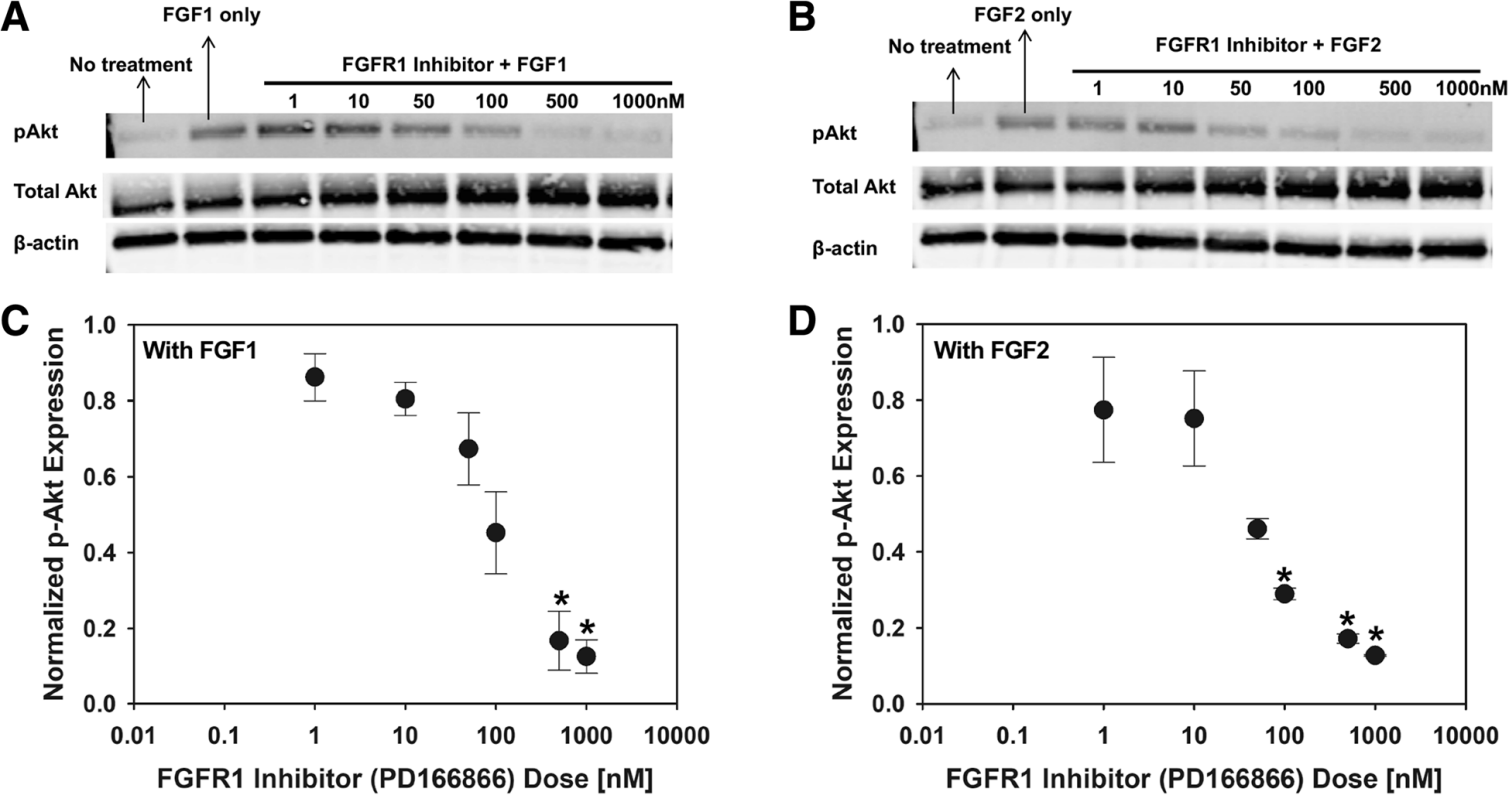
Representative western blot for FGF1 (**a**), and FGF2 (**b**) treated VICs. Semi-quantitative analysis of Akt phosphorylation for FGF1 (**c**), and FGF2 (**d**) treated VICs. *n* = 3, *p < 0.05 compared to samples without FGFR1 inhibitor (PD166866)

In the next series of experiments, we targeted the FGFR1 receptor subtype, based on our IHC results in which FGFR1 was expressed in diseased valve tissues together with the ligands FGF1 and FGF2 (Fig. 3). We used PD166866, a selective FGFR1 inhibitor [21], to study the involvement of FGFR1 in FGF1- and FGF2-mediated mechanosensitive VIC behavior. The appropriate working concentration of PD166866 was determined via western blotting and MTT assay on VIC monolayers. As shown in the western blotting results (Fig. 5a – d), PD166866 effectively inhibited FGF1- and FGF2-mediated Akt phosphorylation in VICs. With FGF1 stimulation (Fig. 5a, c), FGFR1 inhibitor (PD166866) doses of 500 nM and 1000 nM significantly (*p < 0.05) reduced phosphorylated Akt expression compared to samples not treated with FGFR1 inhibitor. With FGF2 stimulation (Fig. 5b, d), FGFR1 inhibitor (PD166866) doses of 100 nM, 500 nM and 1000 nM significantly (*p < 0.05) reduced phosphorylated Akt expression compared to samples not treated with FGFR1 inhibitor. Untreated samples showed similar expression of phosphorylated Akt as in samples treated with the highest doses of PD166866 (Additional file 2: Figure S2). MTT proliferation assays further supported the above dose-response study (Additional file 3: Figure S3). Addition of PD166866 significantly reduced cell proliferation capacity as seen in all samples that had PD166866 treatment as well as no treatment sample (Additional file 3: Figure S3). Based on both western blot and MTT results, we chose 1000 nM of PD166866 as our working concentration for subsequent experiments as it effectively blocked the Akt activation (phosphorylation) caused by FGF1 or FGF2 stimulation.

### FGFR1 inhibition significantly reduced Akt and mTOR phosphorylation in VICs stretched to 10% but not 20% when samples are supplemented with FGF1 or FGF2

**Fig. 6:**
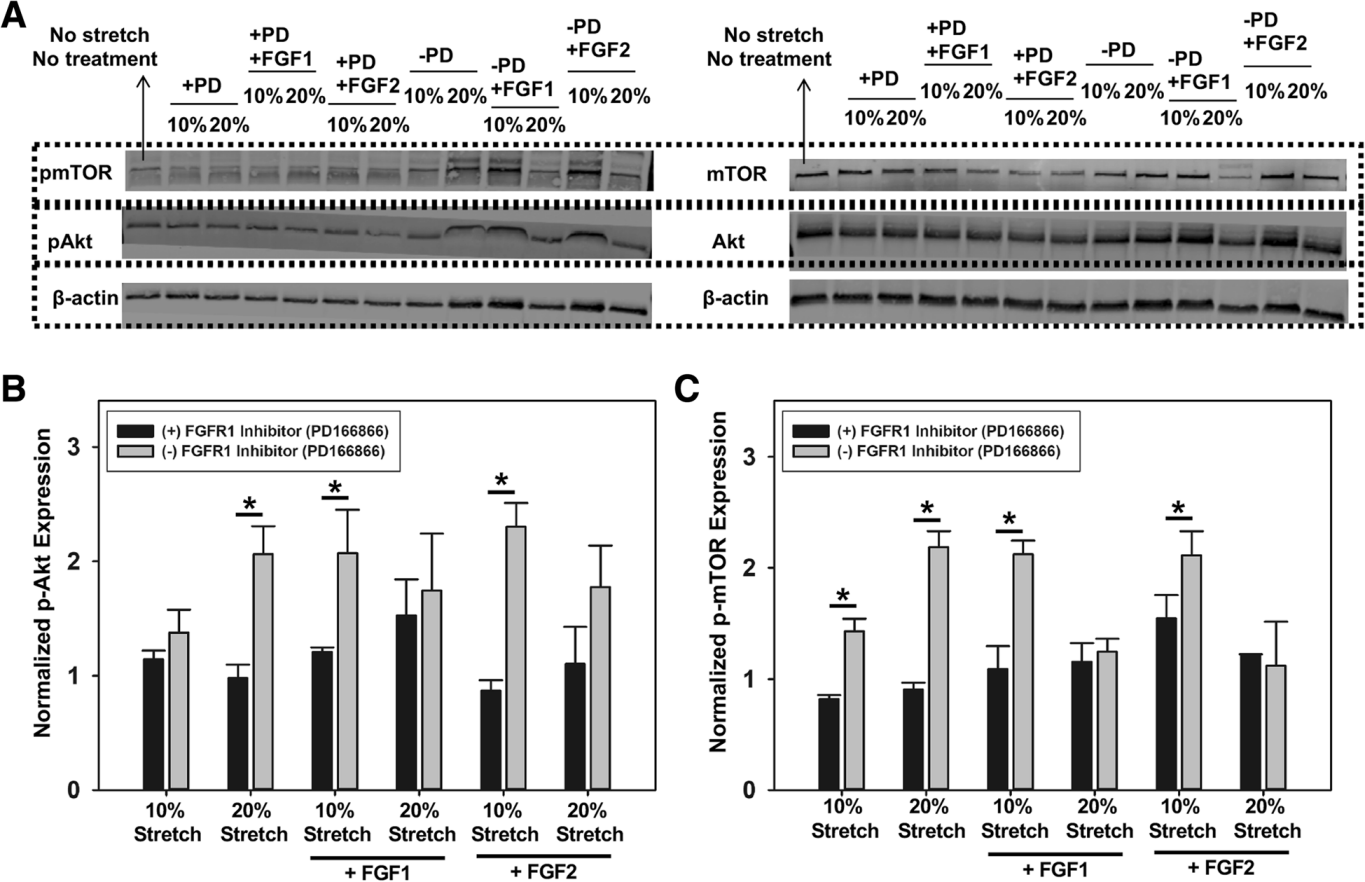
Representative western blot (**a**), and semi-quantitative analysis of Akt (**b**), and mTOR (**c**) phosphorylation under the effects of FGF1/FGF2 and cyclic stretching, *n* = 3, *p < 0.05

To investigate the possible link between FGF-mediated cellular response under stretch stimulation and the Akt and mTOR pathways, we stretched the 3D VIC constructs for 1 h under the previously outlined treatment conditions (Table 2) for western blot analysis (Fig. 6a). Akt phosphorylation was significantly reduced (*p < 0.05) by FGFR1 inhibitor (PD166866) treatment only in VICs stretched to 20% (pathological stretch), and VICs stretched to 10% (normal stretch) when supplemented with FGF1 and FGF2 (Fig. 6b). VICs stretched to 20% (pathological stretch) and supplemented with FGF1 and FGF2 has statistically similar expression levels of phosphorylated Akt in samples with and without FGFR1 inhibitor (PD166866) (Fig. 6b). Similar results were observed for the mTOR phosphorylation experiments (Fig. 6c). FGFR1 inhibition with PD166866 significantly reduced (*p < 0.05) mTOR phosphorylation in VICs stretched to 10% (normal stretch) when samples were supplemented with FGF1 or FGF2, but not in VICs stretched to 20% (pathological stretch). Overall, it appeared that FGF1 and FGF2 supplementation mitigated the activation of the Akt and mTOR pathways of VICs at 20% (pathological) stretch, but not at 10% (normal) stretch.

### Activation of Akt and mTOR pathways correlated with increased VIC metabolic activity and altered phenotype

**Fig. 7:**
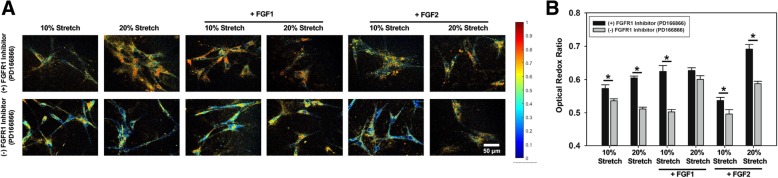
Representative optical redox ratio of VICs (**a**) and corresponding quantitative analysis (**b**); *n* = 5; *p < 0.05

Since the Akt and mTOR pathways directly regulate cellular proliferation [25], we hypothesized that there would be a correlation between FGFR1-mediated activation of the Akt and mTOR pathways with the metabolic activity and proliferative capacity of VICs under different treatment conditions. We have previously shown that optical redox ratio inversely correlated with proliferative potential of VICs [20]. In the current experiments, we observed that optical redox ratio (Fig. 7a) was significantly increased (*p < 0.05) in VICs under all treatment groups in the presence of FGFR1 inhibitor (PD166866) (Fig. 7b).

**Fig. 8:**
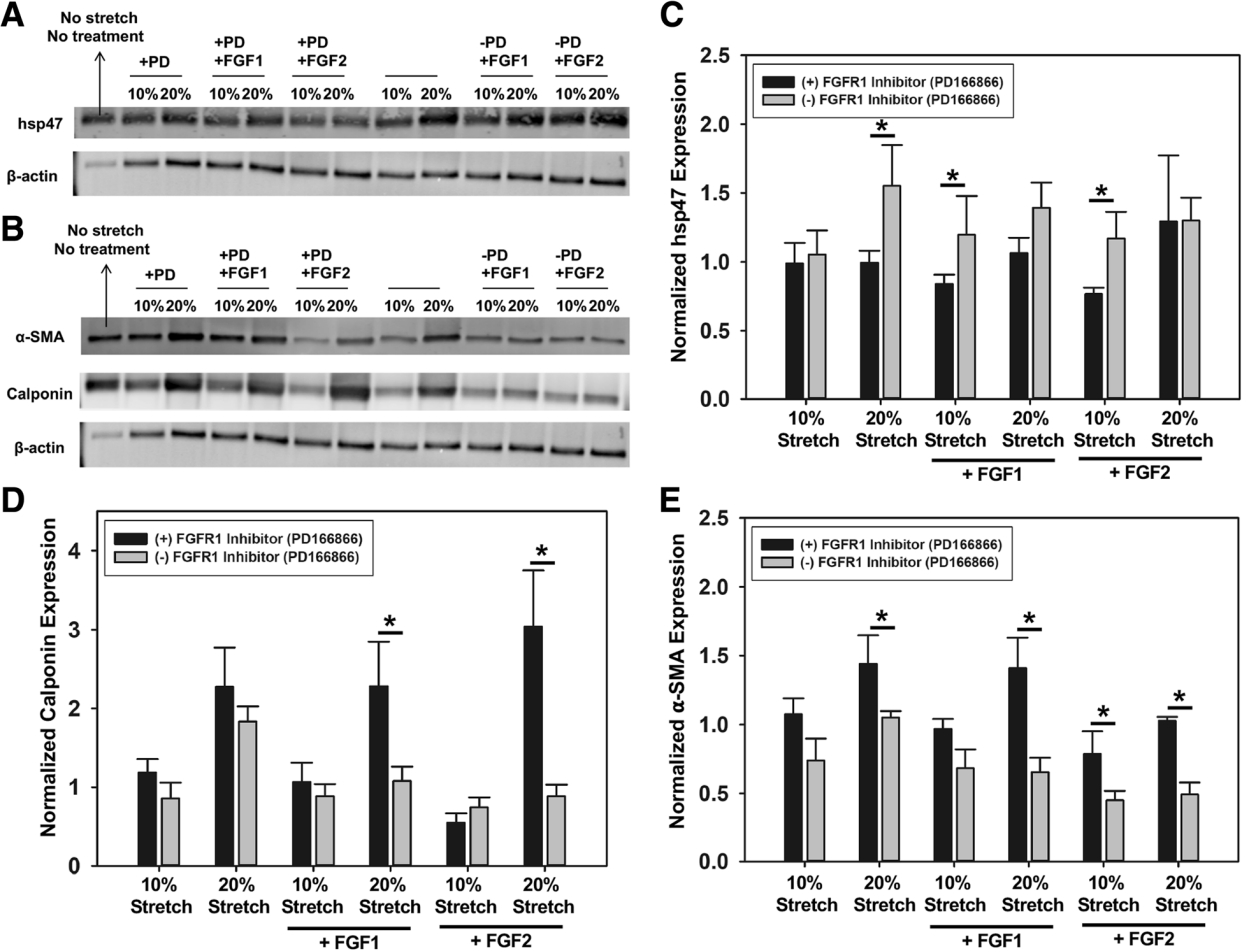
Representative western blots of hsp47 (**a**), α-SMA, and calponin (**b**). Semi-quantitative analysis of hsp47 (**c**), calponin (**d**), and α-SMA (**e**) expression. *n* = 3, *p < 0.05

Western blot analysis (Fig. 8a, b) showed significantly increased (*p < 0.05) hsp47 expression (Fig. 8c) in the absence of the FGFR1 inhibitor (PD166866) in the following treatment groups – VICs stretched to 20% (pathological stretch), and in samples stretched to 10% (normal stretch) when supplemented with FGF1 or FGF2. In terms of the markers for VIC activation, namely, calponin and α-SMA expression (Fig. 8d, e), we observed that FGF1 and FGF2 treatment did maintain VICs from becoming activated at 20% elevated cyclic stretch. Cells at 10% stretch only and cells in 10% stretch with FGF1 or FGF2 treatment had statistically similar expression levels of α-SMA and calponin, suggesting that 10% stretch did not actively induce cell activation and that addition of FGF1 or FGF2 to cells at 10% stretch only affected cell proliferation/metabolism but not cell phenotype. In contrast, cells at 20% stretch had significantly higher α-SMA and calponin compared to cells at 10% stretch and the presence of FGF1 or FGF2 significantly reduced these expression levels. In the presence of FGFR1 inhibitor, all cells that experienced elevated stretch expressed higher amount of α-SMA and calponin.

## Discussion

In vitro cell-tissue culture systems and biomaterials scaffolds, with or without application of mechanical forces, have advanced our understanding of valvular diseases and provided insights into possible intracellular signaling pathways that regulate valve cell pathophysiology [1]. While 2D models are commonly used for growing cells, VICs naturally reside in a three-dimensional (3D) matrix environment. Several pioneering experiments with cells cultured in or on hydrogels comprised of natural ECM proteins or synthetic biomaterials investigated the effect of matrix stiffness on valve cell phenotype. For instance, it was reported that VICs did not form calcified nodules on soft poly (ethylene glycol) (PEG) hydrogels while they had increased expression of calcification markers when cultured on tissue culture polystyrene [26]. Similarly, VICs cultured on soft PEG hydrogel did not adopt a myofibroblast phenotype in response to TGF-β1 treatment [27]. Soft hydrogels were shown to be able to preserve the quiescent phenotype of VICs compared to stiff plastic plates through down-regulation of PI3K/Akt pathway [28]. Although these studies have provided more information about the role of microenvironment signals in regulating valve cell fate, an understanding about how valve cells respond to these cues as well as the possible signaling pathways that mediate cellular responses remains vague. As mentioned above, while most studies in the field are performed on either 2D monolayer cells or ex vivo tissue leaflet explants, cells naturally live in a complex 3D environment. Unfortunately, it is difficult to incorporate any types of mechanical forces that VICs experience in vivo to 3D cell culture system. In this study, we presented a collagen-based scaffold that not only provided cells with 3D architecture but also had sustainable mechanical property. We were able to subject encapsulated VICs to cyclic mechanical stretching condition over the course of 48 h in culture.

The pathophysiological role of the FGF family of ligands on VICs has been an ongoing study in the heart valve field, especially that of FGF2. Several studies have suggested the possible protective role of FGF2 on valve cells under pathological conditions which led to few recent studies where FGF2 was added to cell culture media to maintain VICs in a quiescent state in vitro [10, 11, 29–31]. FGF1 and FGF2 belong to the FGF1 subfamily and they were both expressed in healthy and diseased aortic valve (Fig. 3). It was also reported that FGF1 and FGF2 expression was increased following myocardial infarction in rats [32]. Our results were in agreement with other studies that suggested that FGF1 and FGF2 maintained the quiescent phenotype of VICs as in a native healthy valve [11]. Interestingly, FGF1 and FGF2 not only reduced expression of activated VIC markers, but also appeared to promote increased cell proliferation, compared to cells cultured in 10% FBS-containing media. This data was corroborated by our optical redox imaging results, where cells in media supplemented with FGF had decreased optical redox ratios.

Some of the hallmarks of VIC activation and disease progression, are an increase in proliferation, followed by intensive matrix remodeling, which if not regulated, can result in pathological fibrosis, angiogenesis, chronic inflammation, and eventual calcification [33]. A number of studies have demonstrated that the Akt/mTOR signaling cascade is associated with cardiac hypertrophy [34, 35]. Akt is also involved in regulating the ability of oxidized low-density lipoprotein and lysophosphatydylcholine in the upregulation of ECM protein production in human aortic valve interstitial cells [36]. Accumulation of ECM proteins may contribute to the mechanism of valvular sclerosis associated with the development and progression of aortic stenosis [36]. PI3K/Akt signaling was reported to modulate the NF-ĸB pathway and its downstream target IL-6 which in turn affected the calcification process of VICs [37]. The Akt/mTOR pathways, however, have not been thoroughly examined in the heart valve context, especially under the effect of FGF1 and FGF2 in a 3D stretching model. Our data suggested that FGF1/2-mediated Akt/mTOR signaling activation was dependent on stretch magnitude and correlated with cellular optical redox ratios. Specifically, VICs that had higher level of phosphorylation of Akt/mTOR also displayed decreased optical redox ratios and vice versa. These effects were eliminated in the presence of FGFR1 inhibitor. The optical redox ratio metric has recently been used to assess the metabolic changes in cells during disease initiation and progression. Our previous study on single VICs reported that as cells became more elongated, into a shape that mimicked pathological stretch, their optical redox ratio was reduced which was associated with increased cell proliferation [20]. Other studies reported a decrease in redox ratio in cells undergoing proliferation [38], and osteogenic differentiation [39] or an increase in redox ratio after induction of cell death [40]. Most notably, similar trends in the Akt/mTOR pathways and optical redox ratios were observed in cancer [41, 42].

Overall, it appeared that VICs responded to elevated stretch by activating the Akt/mTOR pathways which increased cellular metabolic activity and proliferative capacity. The treatment of FGF1 and FGF2 for cells under 20% stretch reversed those processes in which both phosphorylation and proliferation were reduced. In contrast, when VICs experienced physiological 10% stretch, they did not activate Akt/mTOR pathway but the addition of FGF1 and FGF2 significantly enhanced cell proliferation. These results suggested that FGF1 and FGF2 promoted cell proliferation physiologically while reducing cell proliferation in elevated cyclic stretch condition. It is worth mentioning that these acute responses were observed only in the duration of 48 h since the seeding time. Future studies may need to prolong the experimental duration to study longer-term responses such as that of collagen remodeling.

VICs are known for their ability to become activated in response to pathological injury or abnormal hemodynamic/mechanical stretch. Activated VICs usually had increased expression of activated markers, α-SMA and calponin, while showing low expression of vimentin [33]. In our study, we observed that after treatment with FGF1 and FGF2, regardless of the stretch magnitude (i.e 10, 20%), cells expressed lesser activated markers (i.e. 10% FGF1, 20% FGF1, 10% FGF2 and 20% FGF2 samples). In the presence of FGFR1 inhibitor, the effect of stretching became more pronounced as cells expressed more activated markers at elevated cyclic stretch. Additionally, VICs proliferated in 20% stretched sample but decreased proliferation when treated with FGF1 and FGF2, suggesting that the presence of FGF1/FGF2 somehow ‘signaled’ the cells and reduced its proliferation propensity at least in the early phase of experiencing abnormal mechanical stretch. In the absence of FGF or in the presence of FGFR1 inhibitor, the effects of stretching became dominant as cells appeared to become activated and proliferated more at elevated stretch as compared to no stretch and 10% stretch.

Throughout the experiments, we could not detect any difference between the effect of FGF1 versus FGF2 on VICs. It was, however, interesting to note that although FGF1/FGF2 appeared to have similar effects on VICs in both monolayer and 3D culture model but its expression pattern was different in healthy versus calcified valves. Whether this suggested that there may be a need for a more complex cell culture model to closely mimic natural heart valve architecture and environment for teasing out the role of FGF1 and FGF2, or longer culture durations, may require further investigation.

## Conclusions

We report here the possible involvement of FGF-mediated signaling in valve cell responses under mechanical stretch via the Akt and mTOR pathways. In our experiments, it appeared that FGF1 and FGF2 could modulate VIC phenotype under different mechanical stimulation conditions. VICs appeared to be reduce expression of activated markers, with high proliferative property in the presence of FGFs. When experiencing elevated 20% stretch, VICs increased proliferation possibly as a compensatory response against extra load. When FGFs were added, the cellular response was to reduce proliferation in the elevated stretch case. Cell proliferation also was observed to associate with Akt and mTOR pathway activation, increased cell metabolism and altered cell phenotype. Overall, this study provided fundamental information about how valve cells behave under abnormal stretch. Future studies need to investigate these phenomena at longer culture durations to get a more complete picture of VIC mechanoresponses in the context of FGF1 and FGF2 signaling.

## Additional files

Additional file 1: **Figure S1.** Quantitative analysis of (A) Ki67 cell proliferation immunolabeled VICs. (B) Plot of Ki67 proliferation vs. VIC optical redox ratio suggesting a negative relation between these quantities (r=-0.981, *p*=0.12). n=5, *p<0.05. (TIF 108 kb)

Additional file 2: **Figure S2.** Western blot (A) and semi-quantitative analysis (B) of Akt phosphorylation of VICs treated with inhibitor (PD166866) or FGF1/2 treatment only. n=3, *p<0.05. (TIF 91 kb)

Additional file 3: **Figure S3.** Quantitative results from MTT assay for (A) FGF1-treated, (B) FGF2-treated, and (C) control treated VICs. n=3, *p<0.05 compared to all other treatment groups. (TIF 152 kb)

## Abbreviations

5HTR2A: 5 Serotonin receptor 2A
5HTR2B: Serotonin receptor 2B
BSA: Bovine serum albumin
DAPI: 4′,6-diamidino-2-phenylindole
DMEM: Dulbecco’s modified eagle media
DMSO: Dimethyl sulfoxide
ECM: Extracellular matrix
FAD: Flavin adenine dinucleotide
FBS: Fetal bovine serum
FGF1: Fibroblast growth factor 1
FGF2: Fibroblast growth factor 2
FGFR: Fibroblast growth factor receptor
Hsp47: Heat shock protein 47
MTT: (3-(4, 5-dimethylthiazolyl-2)-2, 5-diphenyltetrazolium bromide
NADH: Nicotiamide adenine dinucleotide
PEG: Polyethylene glycol
PFA: Paraformaldehyde
PMT: Photomultiplier
PVDF: Polyvinylidene difluoride
TGF-β1: Transforming growth factor-β1
VIC: Valvular interstitial cells
α-SMA: α-Smooth muscle actin
