# Supplementary figures and images for: The role of fibroblast growth factor 1 and 2 on the pathological behavior of valve interstitial cells in a three-dimensional mechanically-conditioned model
Source: J Biol Eng. 2019 May 27;13:45. doi: 10.1186/s13036-019-0168-1 (PMC6537403; doi:10.1186/s13036-019-0168-1)

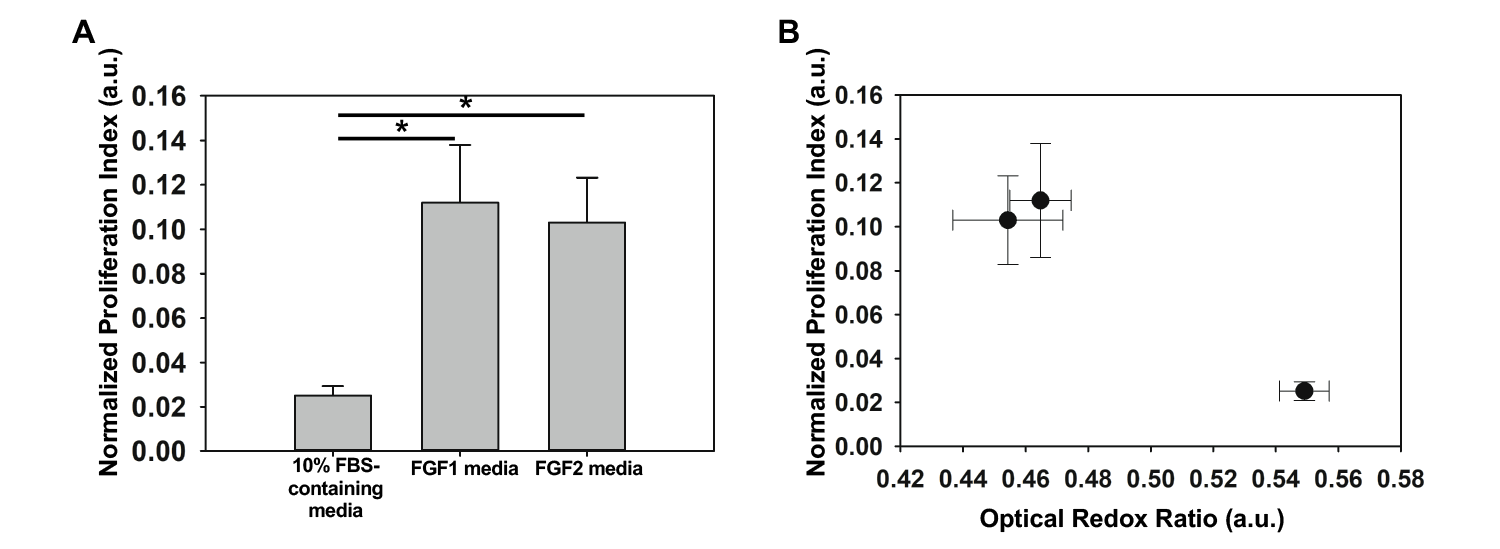

Supplement: Supplementary file 1 — Figure S1. Quantitative analysis of (A) Ki67 cell proliferation immunolabeled VICs. (B) Plot of Ki67 proliferation vs. VIC optical redox ratio suggesting a negative relation between these quantities (r=-0.981, p=0.12). n=5, *p<0.05. (TIF 108 kb) [file 13036_2019_168_MOESM1_ESM.tif]

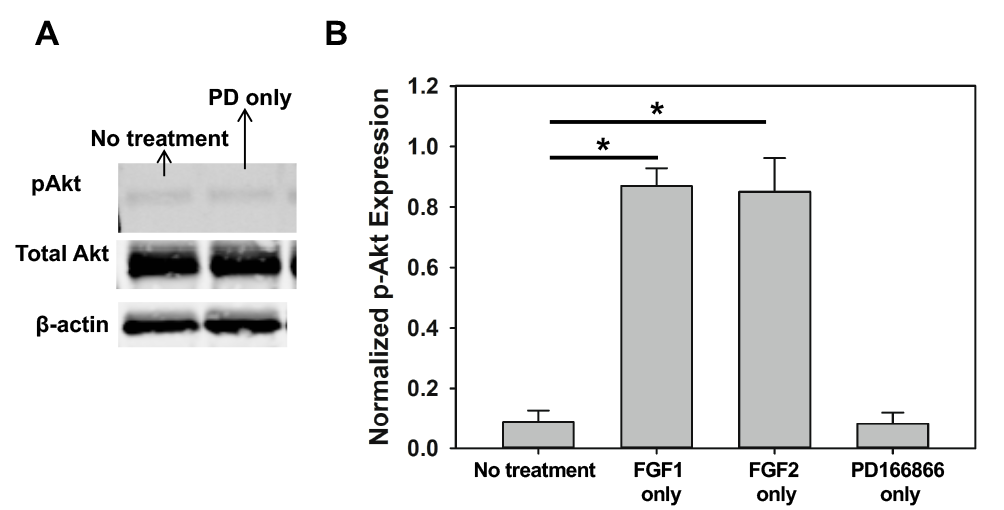

Supplement: Supplementary file 2 — Figure S2. Western blot (A) and semi-quantitative analysis (B) of Akt phosphorylation of VICs treated with inhibitor (PD166866) or FGF1/2 treatment only. n=3, *p<0.05. (TIF 91 kb) [file 13036_2019_168_MOESM2_ESM.tif]

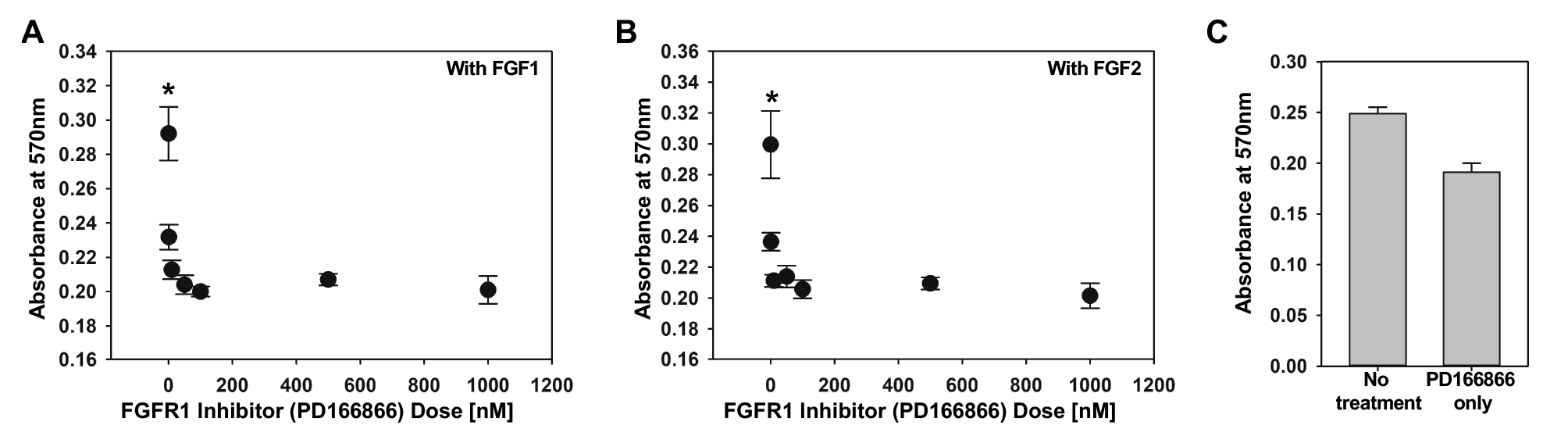

Supplement: Supplementary file 3 — Figure S3. Quantitative results from MTT assay for (A) FGF1-treated, (B) FGF2-treated, and (C) control treated VICs. n=3, *p<0.05 compared to all other treatment groups. (TIF 152 kb) [file 13036_2019_168_MOESM3_ESM.tif]
